# Impact of home-based aerobic training combined with food orientation on food consumption, daily physical activity and cardiorespiratory fitness among breast cancer survivors: six-month clinical trial

**DOI:** 10.1590/1516-3180.2020.0658.28012021

**Published:** 2021-05-10

**Authors:** Andréa Dias Reis, Luciana Sato de Lima, Êmili Amice da Costa Barros, Josefina Bertoli, Luís Alberto Gobbo, Camila Buonani da Silva, João Batista Santos Garcia, Ismael Forte Freitas

**Affiliations:** I PhD. Physical Education Professional, Postgraduate Program on Movement Sciences, Universidade Estadual Paulista (UNESP), Presidente Prudente (SP), Brazil.; II MSc. Dietitian, Postgraduate Program on Physiotherapy, Universidade Estadual Paulista (UNESP), Presidente Prudente (SP), Brazil.; III MSc. Physiotherapist, Postgraduate Program on Physiotherapy, Universidade Estadual Paulista (UNESP), Presidente Prudente (SP), Brazil.; IV MSc. Physical Education Professional and Doctoral Student, Postgraduate Program on Movement Sciences, Universidade Estadual Paulista (UNESP), Presidente Prudente (SP), Brazil.; V PhD. Physical Education Professional and Associate Professor, Postgraduate Program on Movement Sciences, Universidade Estadual Paulista (UNESP), Presidente Prudente (SP), Brazil.; VI PhD. Physical Education Professional and Assistant Professor, Department of Physical Education, Universidade Estadual Paulista (UNESP), Presidente Prudente (SP), Brazil.; VII MD, PhD. Physician and Associate Professor, Postgraduate Program on Adult Health and Postgraduate Program on Health Sciences, Department of Medicine, Universidade Federal do Maranhão (UFMA), São Luís (MA), Brazil.; VIII PhD. Physical Education Professional, Postdoctoral Researcher and Adjunct Professor, Postgraduate Program on Movement Sciences and Postgraduate Program on Physiotherapy, Universidade Estadual Paulista (UNESP), Presidente Prudente (SP), Brazil.

**Keywords:** Breast neoplasms, Exercise, Health, Breast cancer, Physical activity, Women’s health

## Abstract

**BACKGROUND:**

Anticancer treatment gives rise to adverse effects such as increased pain and changes to body weight and menstrual cycles, with negative effects on activities of daily living.

**OBJECTIVE:**

To analyze the effect of food orientation combined with supervised (face-to-face, FF) versus home-based (HB) aerobic training on lifestyle (food consumption and daily physical activity (PA) levels), body composition, metabolic profile and cardiorespiratory fitness, among breast cancer survivors.

**DESIGN AND SETTING:**

Clinical trial study (six months) conducted at a public university in Presidente Prudente, Brazil.

**METHODS:**

Twenty-three female breast cancer survivors (40-75 years old) were allocated to aerobic training groups, either FF or HB. Both groups were trained and received food orientation. They were evaluated through a dietary record, ergometric treadmill test and blood test and the Baecke questionnaire.

**RESULTS:**

After six months, both groups had reduced their lipid levels, total energy consumption and polyunsaturated fat intake, and had increased their PA levels and treadmill test durations. However, only the HB showed reduced carbohydrate percentage and increased folic acid; and only the FF showed reduced lipid, saturated fat and sodium levels, along with increased carbohydrate and protein levels. No differences in body composition or metabolic profile were found.

**CONCLUSIONS:**

These results demonstrated the feasibility of HB aerobic training. In isolation, HB showed greater regulation of carbohydrate percentage and increased folic acid levels. Moreover, these breast cancer survivors presented improvements in food consumption, PA levels and cardiorespiratory fitness, while also maintaining their body composition and metabolic profile after the intervention, independent of the group.

## INTRODUCTION

Hormone therapy is an adjuvant treatment used by breast cancer patients to reduce the risk of tumor recurrence through hormonal blocking, among those with positive hormonal receptors.^1^ Among the different types of hormone therapy, aromatase inhibitors and tamoxifen are the ones most frequently applied to breast cancer patients. Aromatase inhibitors are considered to be more efficient than tamoxifen among menopausal women. Although there is clinical evidence to support the use of hormone therapy, a variety of adverse effects among patients have been reported, such as bodyweight changes, menstrual cycle alterations, pain and risk of uterine cancer.^2^

On the other hand, food orientation allied with physical activity (PA) has been demonstrated to improve quality of life among breast cancer survivors.^3^ Combination of these two interventions has also been found to be advantageous with regard to cancer patients’ body composition and metabolic profile.^4^ These results were obtained through home-based interventions. Recently, this type of intervention was used among breast cancer patients.^4^ Nevertheless, it is less commonly used among patients who are undergoing hormone therapy.

Home-based interventions can be developed in different areas and places, such as public squares and patients’ homes. Hence, this approach has also been used to increase adherence.^5^ Moreover, technology has enabled monitored home-based training sessions.^6^ However, previous studies that involved home-based training did not provide good descriptions of protocol progression and control over training intensity within this method.^3^^,^^4^ Our study contributes to this information, and also compares use of the same protocol in a face-to-face group.

Aerobic training has been the form of exercise most recommended for breast cancer patients, due to its safety.^7^ Furthermore, it can alter their metabolism, as demonstrated through a study on fat mass reduction and lower-limb lean mass increments among 38 obese women who were breast cancer survivors. These women performed three months of aerobic training in which they walked more, did static stretching and were invited to do moderate-intensity exercise at home.^8^

Conversely, some studies are still showing controversial results. In one such study, 22 overweight or obese black breast cancer survivors who underwent an aerobic intervention combined with nutritional counseling for 12 weeks did not show any body weight reduction.^9^ These results demonstrate that there is a lack of consensus in the literature about prescription of aerobic training to improve the metabolic profile and reduce the bodyweight of breast cancer patients. However, aerobic training increases the level of physical activity and cardiorespiratory capacity.^9^ Although the effect of home-based training among patients with breast cancer has been already researched, little has been explored regarding the effect on women undergoing hormone treatment.

## OBJECTIVE

The aim of this study was to analyze the effect of food orientation combined with face-to-face versus home-based aerobic training on lifestyle (food consumption and daily physical activity level), body composition, metabolic profile and cardiorespiratory fitness among women undergoing hormone therapy for breast cancer.

## METHODS

### Subjects

Twenty-three women without current aerobic training who were undergoing hormone therapy (either still in treatment or with treatment concluded) for breast cancer, consisting of use of either tamoxifen or aromatase inhibitors, volunteered to participate in this study. The women who had concluded their treatment had had at least five years of hormone therapy. To be included, the participants needed to have not had any aerobic training for at least three months before the intervention. Potential participants were excluded if they presented any mental disorder or physical condition that would not allow them to perform the aerobic training. In addition, any women who were incapable of verbal communication or had physical impairments or were pregnant or breastfeeding at that time were also excluded. Participants were also excluded if they withdrew from the training or suffered any physical injuries. The women were allocated either to a face-to-face training group (FF) (n = 10) or to a home-based training group (HB) (n = 13).

This study was approved by the Research Ethics Committee of the School of Science and Technology, São Paulo State University (Universidade Estadual Paulista “Júlio de Mesquita Filho”, UNESP), in Presidente Prudente, Brazil, under protocol number 78971417.9.0000.5402 (approved February 26, 2018), and was registered at clinicaltrials.gov under the number NCT03494400 (April 11, 2018). All the procedures were conducted in accordance with the Declaration of Helsinki. Thus, potential participants were informed of the aim and procedures of the study, with a complete explanation, and those who agreed to participate signed a written informed consent statement.

To recruit potential participants, phone numbers of female breast cancer patients who were undergoing hormone therapy were obtained from cancer hospitals. Social media were also used to publicize the study, in order to facilitate inclusion of women living in nearby municipalities or who were not on the phone lists. All the participants were blindly coded and allocated 1:1 with sequence boundaries in the random sequence generator random.org.^10^ The sample size was calculated using statistical software (G-power 3.1; Düsseldorf, Germany). This revealed that with eight participants, a medium effect size of 0.60 would be achieved,^11^ with α error probability of 0.05, power (1-β error probability) of 0.8 and correlation of 0.5, in repeated-measurement analysis of variance (ANOVA) on within-between interaction; and with non-sphericity correction of one, for two groups. There were two analyses over the course of the study.

### Outcomes

The participants’ clinical characteristics were verified at the baseline through an anamnesis. All evaluations were carried out before and after the 24-week intervention.

### Lifestyle

The participants’ nutritional status was determined through a dietary record, which was applied on three occasions: on two workdays and on one weekend day. This dietary record, which consisted of recording all food consumed throughout the day, was collected at the baseline and after the 24 weeks of the intervention.^12^ The software Avanutri Revolution Package, version 4.0 (Avanutri Informatics Ltda, Rio de Janeiro, Brazil) was used to calculate the dietary record.

In order to evaluate the daily physical activity (PA) level, the Baecke questionnaire was applied. This questionnaire has been validated for the Brazilian adult population.^13^ It contains three domains, but the working PA domain was not used in this study since most of the participants were retired or had stepped down due to their health condition.^14^^,^^15^

### Body composition

Anthropometric measurements were made on a mechanical scale (Filizola) to the nearest 0.1 kg; and on a fixed stadiometer (Sanny) to the nearest 0.1 cm or with a measuring tape (Sanny) to the nearest 0.1 cm. The body mass index was also calculated.^16^

A bioelectrical impedance device (BIA Analyzer, Nutritional Solutions, Harrisville, MI, United States) was used to obtain resistance and reactance data. The participants received recommendations regarding clothing, food and drug use, to be evaluated.^17^^,^^18^ The bioelectrical impedance analyzer data were used to calculate fat free mass (kg).^19^ The percentage of fat mass was also calculated.^19^ For the purposes of the present study, obesity was defined as a fat mass percentage ≥ 25%.^20^

### Metabolic profile

The participants’ metabolic profile was analyzed by assaying their glycemia, insulin, triglyceride, total cholesterol and cholesterol fraction levels. For this, blood samples were collected in vacuum tubes with gel without anticoagulant. An enzymatic colorimetric kit was used and the samples were processed in an Autohumalyzer A5 device (Human Gesellschaft für Biochemica und Diagnostica mbH, Wiesbaden, Germany).^21^ The participants were recommended to fast for 12 hours before the blood test, which was done at a specific laboratory (Unilab, Presidente Prudente, São Paulo, Brazil). The metabolic profile was classified as normal or altered.^22^

### Cardiorespiratory capacity

The modified Naughton test was performed on an ergometric treadmill (Inbramed ATL 2000; Inbrasport, Porto Alegre, Brazil), with a maximum capacity of 180 kg, inclination of 26% and velocity of 24 km/h, in a climate-controlled laboratory. This ramp test involved incremental increases in velocity and inclination, and each stage was maintained for two minutes.^26^ The test was ended when the participant reached 85% of her maximum heart rate, as predicted for her age, or when she showed signs for which the test needed to be halted, or when she asked for the test to be stopped because of fatigue.^26^

### Aerobic training

The aerobic intervention combined with food orientation lasted for 24 weeks. The aerobic training followed a recommended duration of 150 minutes per week.^23^ Nonetheless, progressively higher loads were used in order to achieve physiological and metabolic adaptations.^24^

The aerobic training protocol for the FF was undertaken on an ergometric treadmill (Movimento, LX-160, Equipamento de Fitness, Pompeia, São Paulo, Brazil) in an air-conditioned laboratory. The participants trained three times a week on non-consecutive days (Monday, Wednesday and Friday). The intensity of the training was controlled using a heart rate monitor (HR102; Oregon Scientific, São Paulo, Brazil) and was adjusted for the maximum heart rate (HR_max_), according to age (220-age).^25^^,^^26^ The degree of perceived exertion was also recorded at the end of each training session, in order to assess the intensity of the training.^26^ Over the course of the intervention, the participants went through an adaptation process and three progressive stages (Table 1).

**Table 1.** Aerobic training protocol for the supervised (face-to-face) group

Stages	Weeks	Session duration (minutes)	Intensity (% HRmax)	Rate of perceived exertion
Adaptation	1-2	30	50 to 60	11-13 (slight to somewhat heavy)
Stage 1	3-4	40	50 to 60	11-13 (slight to somewhat heavy)
Stage 2	5-12	50	60 to 70	13-15 (somewhat heavy to heavy)
Stage 3	13-24	50	70 to 89	15-17 (heavy to very heavy)

HR_max_ = maximum heart rate.

The aerobic intervention for the HB was undertaken in a variety of environments. These included places near the participants’ homes, public squares and/or inside their homes.^6^ The intensity of each training session and the controlled adjustments to the aerobic training were monitored using the rate of perceived exertion^27^ and HR_max_.^28^ The same protocol as used for the FF group to increase the intensity of the training was also used for the HB group (Table 1).

The participants were encouraged to walk or run at least three times per week. They were also monitored and followed up every 14 days by an exercise specialist from a laboratory. The distance walked or run was verified using a specific application (Google Maps GPS; or Runtastic, Pasching, Austria, 2009).

### Food orientation

Both FF and HB received dietary recommendations for breast cancer survivors,^7^^,^^29^^,^^30^ before the intervention began and in each month of the intervention, provided by a specialized nutritionist. The nutritionist gave information on how to choose good-quality economic foods and on how to prepare and adapt meals according to their energy value and nutrient, fiber and vitamin content, through cellphone communication and in-person meetings.

### Statistical analysis

Descriptive statistics were calculated: means, confidence intervals and absolute and relative frequencies. Data normality and variance homogeneity were verified through the Shapiro-Wilk and Levene tests, respectively. The participants’ characteristics were analyzed using Student’s t test for body mass index and Fisher’s exact test for menopause status, medications, chemotherapy and/or radiotherapy utilization, cancer recurrence and/or metastases, time and type of hormone therapy used, with comparisons between FF and HB.

Repeated ANOVA measurements (within-between interactions) were calculated to assess food consumption, daily PA level, body composition and metabolic profile. Subsequently, the Bonferroni post-hoc test was used to verify any statistical differences. The chi-square test and Fisher’s exact test were used to verify body composition and metabolic profile classification at the baseline and after the 24 weeks of the intervention. The chi-square test was used to analyze total cholesterol, low-density lipoprotein-cholesterol (LDL-C), non-high-density lipoprotein-cholesterol (non-HDL-C), triglycerides and glycemia; while Fisher’s exact test was used to analyze HDL-C and insulin. The effect size was calculated by means of ηp^2^. The SPSS 21.0 software (SPSS Inc., Chicago, IL, United States) was applied to perform the statistical analysis. A statistical difference was considered to be present when P < 0.05.

## RESULTS

**Figure 1.**
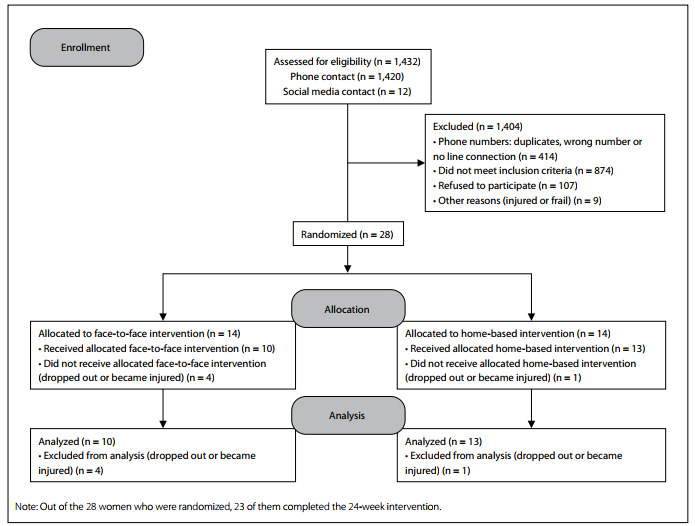
Consolidated Standards of Reporting Trials (CONSORT) flow diagram.

Twenty-eight women undergoing hormone therapy for breast cancer participated in this study, and 23 of them completed the 24-week intervention (Figure 1). It was observed that the participants in the FF and HB groups were similar at the baseline. Nevertheless, the type of hormone therapy used differed between the groups (P = 0.036) (Table 2). All the participants had undergone breast cancer surgery.

**Table 2.** Anthropometric and clinical characteristics among the breast cancer survivors

Variables	Face-to-face group (n = 10)	Home-based group (n = 13)	P-value
Age (years)	61.91 (56.87-66.95)	55 (50.27-60.23)	0.050
Body mass index (kg/m^2^)	30.98 (27.53-34.42)	29.17 (26.71-31.63)	0.348
Menopause, n (%)			
30-39 years	2 (18.2)	0	0.549
40-49 years	6 (54.5)	8 (66.7)	
≥ 50 years	3 (27.3)	3 (25)	
No information	0	1 (8.3)	
Treatment combinations completed			
Chemotherapy + radiotherapy	4 (36.4)	6 (50)	0.680
Without combination	7 (63.6)	6 (50)	
Chemotherapy completed			
Yes	6 (54.5)	7 (58.3)	1.000
No	5 (45.5)	5 (41.7)	
Radiotherapy completed			
Yes	7 (63.6)	10 (83.3)	0.371
No	4 (36.4)	2 (16.7)	
History of cancer recurrence or metastasis			
Yes	1 (9.1)	3 (25)	0.590
No	10 (90.9)	9 (75)	
Hormone therapy phase			
Treatment	6 (54.5)	11 (91.7)	0.069
Concluded	5 (45.5)	1 (8.3)	
Hormone therapy type			
Aromatase inhibitor	8 (72.7)	4 (33.3)	**0.036**
Tamoxifen	2 (18.2)	8 (66.7)	
Used both	1 (9.1)	0	

Values are expressed either as mean (confidence interval) or as absolute frequency (relative frequency).

Statistical tests: Student’s t test (age and body mass index) or Fisher’s exact test (menopause, treatment combinations, chemotherapy, radiotherapy, cancer recurrence or metastasis and hormone therapy and type of hormone therapy); significance level: P-value < 0.05*.*

We observed that the participants’ lipid consumption (g) presented reductions, as shown in the post-hoc test: for FF, P = 0.001; and for HB, P = 0.008. The percentage of lipid consumption decreased only for FF (P = 0.002). The carbohydrate percentage presented interaction (f = 7,216; P = 0.023; ηp^2^ = 0.419) and difference in the time analysis (f = 5.012; P = 0.049; ηp^2^ = 0.334), with increased levels in FF (P = 0.007). The groups differed at the baseline (P = 0.022) (Table 3).

**Table 3.** Food consumption among breast cancer survivors who underwent aerobic training combined with food orientation

Variables	Face-to-face group (n = 10)		Home-based group (n = 13)					ηp^2^
	Baseline	24 weeks	Baseline	24 weeks	Time	Group	Interaction	
Total energy consumption (kcal)	1960.20 (1606.81-2313.58)	1583.36 (1327.61-1839.11)^#^	1982.75 (1780.18-2185.32)	1634.27 (1418.40-1850.13)^#^	**0.001**	0.842	0.842	0.694
Carbohydrates (g)	50.32 (46.19-54.46)*	59.09 (54.14-64.03)^#^	57.30 (51.70-62.90)	57.38 (51.78-62.98)	**0.049**	0.353	**0.023**	0.334
Lipids (g)	67.79 (50.03- 85.55)	34.74 (22.55-46.93)^#^	57.36 (44.99-69.72)	43.10 (28.70-57.51)^#^	**< 0.001**	0.908	**0.039**	0.761
Protein (g)	94.56 (75.74-113.39)	86.08 (70.76-101.40)	82.89 (70.85-94.94)	79.63 (67.08-92.19)	0.298	0.413	0.506	0.108
Carbohydrates (%)	242.96 (205.46-280.46)	231.60 (197.18-266.02)	283.74 (244.80-322.68)	231.95 (201.67-262.23)^#^	**0.025**	0.362	0.089	0.409
Lipids (%)	30.27 (25.31-35.24)	19.11 (13.99-24.24)^#^	25.78 (21.15-30.40)	23.00 (17.19-28.82)	**0.002**	0.913	**0.013**	0.616
Protein (%)	19.40 (17.52- 21.29)	21.80 (19.54-24.07)^#^	16.92 (14.26-19.58)	19.61 (17.14-22.09)	**0.020**	0.139	0.863	0.435
Saturated fat (g)	27.70 (18.79-36.60)	15.31 (8.50-22.13)^#^	23.64 (17.79-29.48)	19.58 (15.14-24.01)	**0.003**	0.980	0.137	0.605
Polyunsaturated fat (g)	18.41 (11.32-25.49)	9.71 (4.33-15.09)^#^	14.45 (10.71-18.18)	10.90 (6.60-15.20)^#^	**0.004**	0.697	0.178	0.576
Monounsaturated fat (g)	9.29 (4.58-14.01)	5.60 (2.88-8.32)	9.19 (5.30-13.08)	8.68 (5.91-11.45)	0.256	0.196	0.327	0.127
Cholesterol (mg)	265.70 (184.81-346.59)	314.91 (154.37-475.45)	253.10 (146.49-359.71)	181.29 (134.43-228.14)	0.818	0.112	0.201	0.006
Fiber (g)	17.69 (12.43-22.95)	20.87 (18.48-23.26)	16.17 (10.98-21.37)	19.03 (15.31-22.75)	0.179	0.498	0.896	0.172
Protein (g/kg)	1.31 (1.0272-1.5964)	1.18 (0.99-1.36)	1.21 (1.00-1.43)	1.16 (0.95-1.37)	0.256	0.720	0.481	0.127
Sodium (mg)	1671.39 (1004.06-2338.71)	601.73 (405.78-797.69)^#*^	1650.70 (980.25-2321.16)	1226.74 (780.68-1672.80)	**0.003**	0.302	0.246	0.602
Potassium (mg)	1950.06 (1503.57-2396.55)	2017.01 (1659.52-2374.49)	1691.75 (1168.86-2214.64)	1853.23 (1496.25-2210.22)	0.582	0.410	0.723	0.031
Folic acid (mg)	137.65 (83.69-191.60)	170.88 (125.74-216.03)	107.83 (72.01-143.65)	170.09 (139.65-200.53)^#^	**0.014**	0.540	0.330	0.468
Calcium (mg)	664.54 (415.00-914.08)	600.01 (421.36-778.65)	567.33 (417.00-717.66)	517.49 (338.52-696.46)	0.408	0.445	0.887	0.069
Phosphorus (mg)	1126.37 (875.75-1376.99)	1164.84 (988.64-1341.05)	940.83 (760.85-1120.80)	908.63 (762.38-1054.88)	0.953	0.113	0.586	< 0.001
Iron (mg)	17.33 (14.04-20.62)	16.29 (12.66-19.91)	14.61 (11.79-17.42)	13.44 (10.10-16.78)	0.313	0.204	0.965	0.101

Values are expressed as mean (confidence interval). ^#^Statistical difference within group; ^*^statistical difference between groups; statistical test: ANOVA (within-between interactions) and thereafter the Bonferroni post-hoc test; effect size: ηp^2^; significance level: P-value < 0.05*.*

The total energy consumption (kcal) and polyunsaturated fat (g) showed reductions in the FF group (P = 0.003; P = 0.018), as also did HB (P = 0.010; P = 0.047), in the post-hoc test. The FF group presented an increase in protein (%) (P = 0.005) and a reduction in saturated fat (g) (P = 0.016). The sodium level (mg) was influenced over time (f = 15.134; P = 0.003; ηp^2^ = 0.602), with a reduction for FF (P = 0.010), such that there was a difference between the groups at the end of the 24-week intervention (P = 0.027). Conversely, the carbohydrate percentage (P = 0.029) presented a reduction and folic acid (mg) presented an increase (P = 0.018) in HB (Table 3).

We observed increases in physical exercise levels, with regard to the dimensions of both leisure PA and locomotion PA, in both the FF group (P = 0.004; P = 0.024) and the HB group (P = 0.035; P = 0.040) (Table 4). The duration of the treadmill test showed a difference in the time analysis (f = 10,811; P = 0.008; ηp^2^ = 0,519), with different increases in FF (P = 0.010) and in HB (P = 0.038) (Table 5).

**Table 4.** Daily physical activity level, body composition and metabolic profile among breast cancer survivors who underwent aerobic training combined with food orientation

Daily physical activity level	Face-to-face (n = 10)		Home-based (n = 13)					ηp^2^
	Baseline	24 weeks	Baseline	24 weeks	Time	Group	Interaction	
Exercise and leisure physical activity	2.18 (1.87-2.49)	2.64 (2.26-3.01)^#^	2.27 (2.07-2.48)	2.61 (2.42-2.80)^#^	**0.004**	0.801	0.472	0.583
Activities of locomotion	1.95 (1.53-2.38)	2.43 (2.06-2.81)^#^	2.02 (1.67-2.38)	2.36 (2.15-2.58)^#^	**0.005**	1.000	0.574	0.566
Cardiorespiratory fitness								
Test time (minutes)	6.36 (4.88-7.84)	7.45 (5.97-8.94)^#^	6.45 (5.49-7.42)	7.18 (5.91-8.45)^#^	**0,008**	0,915	0,307	0,519
Body composition								
Weight (kg)	73.78 (65.55-82.02)	73.66 (64.70-82.63)	69.53 (64.33-74.73)	69.28 (65.11-73.45)	0.765	0.333	0.888	0.009
Waist circumference (cm)	90.14 (83.14-97.13)	91.03 (84.03-98.02)	84.81 (80.14-89.48)	84.31 (80.22-88.40)	0.740	0.192	0.163	0.011
Fat-free mass (kg)	40.18 (37.17-43.19)	40.20 (36.94-43.47)	39.39 (37.63-41.15)	39.32 (37.45-41.20)	0.914	0.616	0.826	0.001
Fat mass (%)	44.96 (42.07-47.84)	44.75 (41.73-47.77)	42.93 (39.45-46.40)	43.02 (40.19-45.85)	0.900	0.240	0.731	0.002
Metabolic profile								
Total cholesterol (mg/dl)	190.82 (172.49-209.15)	189.55 (173.12-205.97)	176.55 (159.25-193.84)	169.55 (150.88-188.21)	0.299	0.177	0.590	0.107
HDL-C (mg/dl)	58.45 (49.05-67.86)	56.09 (49.29-62.89)	57.64 (49.48-65.79)	58.09 (49.78-66.40)	0.551	0.914	0.354	0.037
LDL-C (mg/dl)	107.35 (90.75-123.95)	110.62 (94.14-127.09)	96.89 (80.61-113.17)	89.81 (70.77-108.85)	0.570	0.235	0.249	0.033
Non-HDL-C (mg/dl)	132.36 (114.07-150.66)	133.45 (115.66-151.25)	118.91 (100.40-137.41)	111.36 (92.13-130.59)	0.368	0.210	0.358	0.081
Triglycerides (mg/dl)	139.00 (106.81-171.19)	123.25 (96.11-150.38)	122.00 (91.33-152.67)	120.91 (93.03-148.79)	0.429	0.643	0.152	0.064
Glycemia (mg/dl)	101.03 (89.18-112.88)	107.0364 (95.62-118.46)	94.75 (68.03-121.46)	90.53 (75.67-105.38)	0.827	0.333	0.091	0.005
Insulin (mcIU/ml)	15.69 (11.64-19.74)	18.63 (12.71-24.54)	11.83 (7.47-16.19)	11.79 (8.14-15.44)	0.106	0.136	0.212	0.239

Values are expressed as mean (confidence interval). ^#^Intra-group difference; statistical test: analysis of variance (within-between interactions) and thereafter the Bonferroni post-hoc test; effect size: ηp^2^; significance level: P-value < 0.05*.*

HDL-C = high-density lipoprotein-cholesterol; LDL-C = low-density lipoprotein cholesterol.

**Table 5.** Assessment of metabolic profile in breast cancer survivors who underwent aerobic training combined with food orientation, independent of training groups

Metabolic profile	Baseline		24 weeks		P-value	ηp^2^
	Altered	Normal	Altered	Normal		
Total cholesterol (mg/dl)	10 (43.5)	13 (56.5)	7 (30.4)	16 (69.6)	0.359	0.135
HDL-C (mg/dl)	2 (8.7)	21 (91.3)	1 (4.3)	22 (95.7)	1.000	0.088
LDL-C (mg/dl)	14 (60.9)	9 (39.1)	12 (52.2)	11 (47.8)	0.552	0.088
Non-HDL-C (mg/dl)	17 (73.9)	6 (26.1)	14 (60.9)	9 (39.1)	0.345	0.139
Triglycerides (mg/dl)	8 (34.8)	15 (65.2)	7 (30.4)	16 (69.6)	0.753	**0.046**
Glycemia (mg/dl)	7 (30.4)	16 (69.6)	8 (34.8)	15 (65.2)	0.753	**0.046**
Insulin (mcIU/ml)	1 (4.3)	22 (95.7)	3 (13)	20 (87)	0.608	0.145

Values are expressed as absolute frequency (relative frequency). Statistical test: chi-square test (total cholesterol, LDL-C, non-HDL-C, triglycerides and glycemia) or Fisher’s exact test (analysis on HDL-C and insulin); effect size: ηp^2^; significance level: P-value < 0.05.

HDL-C = high-density lipoprotein-cholesterol; LDL-C = low-density lipoprotein cholesterol.

Body composition and metabolic profile did not present significant differences (Table 4). Nonetheless, the alterations in the metabolic profile, independent of the training group, demonstrated that the majority of the participants were within the normal range at the baseline, except for LDL-C (39.1%) and non-HDL-C (26.1%). It was also observed that after the 24-week intervention, there were more participants with a metabolic profile that was considered normal, except for glycemia and insulin (Table 5).

## DISCUSSION

In this study, the nutritionist’s recommendations were given in person and online via smartphones. It is worth mentioning that in the literature, it has been established that informative interventions combined with aerobic training seem to be more efficient with regard to improving quality of life, compared with interventions alone.^3^

Hence, food orientation seems to be a means for changing habits. On the other hand, changes in dietary habits might also be due to alterations related to physical exercise, which generate improvements in the anti-inflammatory process. Consequently, leptin and insulin signaling in conjunction with the central nervous system improve the signaling of the satiety command in the hypothalamus.^31^

Our results showed that total energy consumption (kcal) decreased, both in FF and in HB. This finding corroborated the data in the literature. A feasibility study among 22 overweight and obese black female breast cancer survivors who participated in an aerobic intervention for 12 weeks found that reductions in energy intake and fat consumption occurred.^9^

Another noteworthy finding in this study was the decreases in macronutrient intake, such as lipid (g) and polyunsaturated fat (g) consumption. These data indicate that our intervention enabled adjustment of fat consumption in both groups and adjustment of lipid consumption in FF.^32^

We observe broader alterations in the FF group, which included simultaneously increased protein (%) and carbohydrate (g) intake, along with reduced lipid (%) and saturated fat (%) levels. However only HB showed a carbohydrate reduction (%). Especially for FF, these results indicated that the balance in macronutrient consumption was enhanced. This was possibly because the adjustments that macronutrients were able to provide to the liver and muscle fat became better. In this regard, overfeeding on sucrose and fat, combined with low protein content, has been seen to increase storage of intrahepatocellular and intramyocellular lipids, consequently increasing fat accumulation in the liver and muscles.^33^

The sodium (mg) consumption was also reduced in both groups, which seems to be a prevention factor against breast cancer recurrence. A cohort study carried out among South African women showed that there was an association between salty foods and the risk of breast cancer development during the post-menopause period.^34^

We observed that in the HB group, the amount of folic acid intake was enhanced. In both groups, folic acid patterns were within normality after the intervention. Folic acid is composed of vitamin B, which is essential for biological reactions since folates provide carbon for deoxyribonucleic acid (DNA) biosynthesis and thus assist in maintaining the gene balance. Furthermore, folate deficiency is associated with cardiovascular and brain diseases, and also cancer.^35^ Folic acid concentrations in the range of 18-32 μmol/l have less association with the risk of development of breast cancer in patients ≤ 50 years old, except in those who carry the gene 677 Ncc.^35^ However, the researchers in that study did not analyze the values in patients with breast cancer.

In the literature, it has been proposed that changes to food habits through aerobic home-based training interventions may be a differential in clinical research. This may be due to changes in consumption of fiber and processed meat, along with better choice of purchases, such that vegetables, whole grains, proteins and legumes are bought instead of sweets and processed snacks.^36^ Therefore, aerobic home-based interventions that are monitored online with nutritional counselling seem to have positive effects on food habits, compared with FF interventions.^36^

In the present study, similar results for FF and HB were observed regarding increases in PA levels and maintenance of body composition and metabolic profile. These results are in accordance with those from a study in which 100 women with breast cancer were assessed. The sample was separated into three groups: one of them received counselling via telephone, another received on-site recommendations and the third was a control group. The two intervention groups received counselling aimed at decreasing their energy intake, and underwent regular aerobic PA combined with behavioral therapy, for six months.^4^ The telephone counselling and on-site groups showed similar results. A significant reduction in body weight was detected, thus differing from the control group, which remained unchanged throughout the intervention.^4^ However, weight reduction was only identified when there was a comparison with the group without intervention.^4^ Conversely, our study did not have a group without intervention, because the goal was to compare intervention models. These results highlight that HB intervention seems to be efficient among women with breast cancer.

Our data indicated that changes in lifestyle occurred, along with increased PA levels and improved food consumption. However, these improvements did not provide body weight loss. The results from this study are in agreement with data in the literature, in which no significant improvements in body composition were found after six months of aerobic intervention and nutritional counselling, among 61 breast cancer women undergoing chemotherapy.^37^ Therefore, it appears that difficulty in reducing body weight and improving body composition, after an aerobic intervention combined with food orientation, is present at different stages of breast cancer.

Regarding the effects of hormone therapy on body weight gain, no significant differences between aromatase inhibitors and tamoxifen were observed in comparison with a placebo.^38^ On the other hand, use of aromatase inhibitors seems to cause less body weight increase than does estrogen receptor selective modulator.^38^ This demonstrates that endocrine agents might not be the only cause of body weight gain, considering that other events may occur concomitantly, such as chemotherapy and menopause. All of these other events may encompass factors related to body weight increase.^38^ Moreover, the aging process could be another factor, given that middle aged women’s body weight increases by about 0.5 kg per year.^39^ In contrast, maintenance of body composition and metabolic profile could be positive for women undergoing hormone therapy for breast cancer, when these variables are within the recommended values.

In this study, most of the participants were within the recommended range of metabolic profile.^22^ This may have influenced maintenance of this parameter without the need for metabolic adjustments caused by the aerobic intervention and food orientation. However, the training volume might not have been efficient with regard to ameliorating the metabolic profile.^24^ Nevertheless, practicing moderate to vigorous PA for ≥ 150 minutes per week has been correlated with better levels of HDL-C and triglycerides.^40^

Clinically, cancer survivors show metabolic alterations, as well as dysfunction or increased cardiac overload, in comparison with individuals of a similar age without a diagnosis of cancer.^41^ In order to test the fitness level of our participants, we used the modified Naughton test, which is recommended for individuals with chronic conditions, older adults and individuals with low physical activity levels.^26^ The duration of the stress test is a parameter for estimating cardiopulmonary capacity.^42^ The results from our study showed that the number of stages completed during the test increased in both groups, which suggests that among women undergoing hormone therapy for breast cancer, not only supervised aerobic training but also a home-based approach can improve cardiorespiratory capacity.

Some limitations of the present study need to be acknowledged. These include prescription of training on the basis of HRmax, which may present variability between participants of a mean of 12 heartbeats/minute.^26^ On the other hand, use of the perceived exertion rate was helpful for the home-based training protocol. Additionally, the sample size was another limitation in this study, owing to the difficulty in recruiting women with breast cancer who were undergoing hormone therapy, in a city of approximately 208,000 inhabitants. Nevertheless, a medium effect size was predominant among the study variables and we were able to investigate the feasibility of an intervention.

We suggest that multicenter studies should be conducted on this population, with analysis on inflammatory cytokines, in order to better comprehend the nutritional alterations caused by the relationships of body composition and metabolic profile with the central nervous system.

## CONCLUSION

The aerobic intervention combined with food orientation was a feasible strategy for promoting improvements in food consumption, daily PA and cardiorespiratory fitness, and for maintaining body composition and metabolic profile, among women undergoing hormone therapy for breast cancer.

The groups also stood out individually, such that in the FF group, there was greater regulation of macronutrients and, in the HB group, the folic acid level increased and the percentage of carbohydrates decreased. These results demonstrated the feasibility of HB aerobic training, as a complementary treatment for breast cancer survivors.
